# Using co-design to create a fit-for-purpose debriefing framework for at-scale healthcare simulation

**DOI:** 10.1186/s41077-026-00441-y

**Published:** 2026-04-30

**Authors:** Michelle A Kelly, David Freer, Lyn Gum

**Affiliations:** School of Nursing and Midwifery, College of Health, Adelaide University, North Terrace, Adelaide, South Australia 5000 Australia

**Keywords:** Simulation-based education, Debriefing frameworks, Context specific, Co-design, Healthcare, Nursing students, At-scale

## Abstract

It is widely acknowledged that debriefing within or following simulation-based education promotes reflection on and about practice. Resuming in-person simulations post-COVID-19 in our educational setting revealed that post-simulation debriefing was lacking. Given the large numbers of students and casual clinical educators we embarked on a project to reintroduce debriefing to simulation activities. While several frameworks exist to guide debriefing in clinical settings, approaches to debriefing undergraduate students in educational settings differ in intent and context and needs to align with local curricula and regulatory standards of practice. Acknowledging the substantial impact to teaching practices of this project, we used an educational co-design approach to maximise staff engagement, ownership and adoption of a local fit-for-purpose debriefing framework. We applied the 5 steps within the educational *Connected Learning at Scale* framework to project activities: explore/ideate; plan/design; build/test; implement/observe and reflect/evaluate. The aim of this paper is to provide details of the processes undertaken in developing an evidence-informed fit-for-purpose tool for use at-scale, engendering engagement and ownership, for context specific implementation. While developed with debriefing for simulation in mind, the localised, fit-for-purpose tool is flexible and appropriate to use for other university learning contexts.

**Supplementary Information:**

The online version contains supplementary material available at 10.1186/s41077-026-00441-y.

## Introduction

Debriefing is repeatedly identified as one of the most important elements in healthcare simulation [1]. Debriefing is a purposeful conversation where learners and facilitators reflect on events within the simulation, identify gaps in performance, explore rationale for behaviours, and distil and solidify key learning points related to practice [2]. In pre-registration nursing curricula, simulation-based education (SBE) is an important strategy for students to rehearse and refine skills and apply their learning to clinical practice [3]. In this context, debriefing can improve and contextualise a wide range of clinical skills while facilitating self-reflection [4] and further promote deep learning by enhancing critical thinking and self-efficacy [5].

Debriefing within higher education settings differs somewhat to the approaches taken in clinical settings where the focus is often on reviewing patient care situations to improve service delivery and reduce morbidity and mortality [6]. SBE within undergraduate nursing curricula pays attention to the relevant program and subject learning outcomes, and focuses on preparation of students for clinical experiences and entry to the workforce [7]. Facilitated debriefing led by experienced nurse clinicians is a preferred approach in many university settings including Australia particularly with novice nursing students, who are in the early stages of developing their professional identity and scope of practice [8].

Debriefing frameworks and tools provide structure to guide the process, potentially improving the quality of debriefing by defining, organising, and describing the purpose of specific aspects of the debriefing conversation [9, 10]. Implementation of debriefing frameworks across a range of settings has been shown to promote facilitator confidence and understanding, improve consistency across learner groups, and enabled greater participation in discussions [4, 11]. For novice debriefers in particular, frameworks provide structure and guidance across the common elements, promoting confidence and autonomy [12, 13]. Further, debriefing that is poorly executed, or without structure or guidance, can negatively impact on learning [1]. The importance of debriefing has also been acknowledged and included in the Healthcare Simulation Standards of Best Practice™ with recommendations that a planned debriefing, based on a contemporary framework, be included within or following simulation experiences [14].

Although numerous healthcare simulation debriefing frameworks have been described in the literature [15–21] attention to local, cultural and discipline specific aspects need further consideration for maximal benefit. For example, discourses, clinical practices, student cohorts and curricula in accredited higher education baccalaureate nursing programs differ from country to country [22, 23]. Many Australian university programs comprise large and diverse nursing cohorts, more than 1,000 domestic and international students per year, providing different challenges in SBE practices. Ensuring meaningful, at scale, SBE experiences particularly with debriefing, requires balancing curricula requirements, class schedules, staffing and providing frameworks that align with local contexts.

During the global COVID-19 pandemic, elements of teaching delivery at universities across Australia and globally were condensed, altered or reconfigured due to restrictions of face-to face teaching and an imperative to ensure adequate numbers of graduates for a depleting workforce [24]. As standard face-to face teaching in our institution resumed in mid-2022 and senior staff experienced in simulation were appointed, attention refocused on the delivery of SBE to ensure that debriefing, in particular, aligned with contemporary practices. As no standard framework was in place at the time, we wanted to implement one that resonated with local clinical nursing and educational practices and contexts, mindful of our diverse student cohorts. The approach chosen to maximise staff engagement and collaboration in the process was co-design [25]. Numerous frameworks exist for co-design, also referred to as co-production or co-creation [26]. Although contexts and application differ, co-design in an educational setting refers to stakeholders co-leading the development, design, implementation and evaluation of activities [26]. Co-creation focuses on identifying a ‘problem’ and creating opportunities for stakeholder engagement from the outset. In our context, co-creation reflected the role and activities of the lead team members scoping workable solutions to the ‘problem’ and practical approaches to gain wider stakeholder opinions and solutions [26]. Co-production on the other hand reflects engagement of stakeholders at the end of a project where acceptability and feasibility of a the ‘end product’ is sought [26].

A key factor in using the co-design approach was the large casual clinical nurse educator workforce of over 70 staff. While overseen by a small group of permanent university nursing clinical educators, variability in approaches to debriefing with students, drawn from staff’s clinical workplaces, was evident. The aim of this project was to mobilise staff knowledge and provide opportunity to co-design a fit-for-purpose, evidence-informed contemporary framework for debriefing large diverse cohorts of nursing students. The co-design approach was chosen to develop and implement positive, sustained teaching practices while optimising learning and increasing performance [27], purposely enabling collaboration with or between multiple stakeholders as contributors [28, 29].

The aim of this paper is to describe the steps taken in this co-design project, the benefits, challenges and lessons learned in the context of SBE debriefing at-scale in a large university nursing program. Through staff workshops, and creation of supportive educational resources, the intent was to implement a sustained and acceptable debriefing approach ensuring equitable student learning experiences.

### An opportunity to improve practice

The impetus for developing a fit-for-purpose debriefing framework was a deficit in debriefing practices in the simulation components of the undergraduate nursing program at our university. Following resumption of all on-campus activities we conducted an audit of SBE practices. The Bachelor of Nursing degree provides education for approximately 3,000 students, with close to 1,000 enrolled in each year of the 3-year program. Additionally, an accelerated 2-year program is offered to within-country and international practicing nurses who wish to attain a Bachelor degree, resulting in diversity of learner needs.

Approximately 70 casual educators who concurrently work clinically, are employed in the simulation labs to facilitate student learning, with oversight from a small, dedicated group of permanent nursing clinical educators. At times, securing staffing for the large number of classes has been challenging, particularly with casual staff’s work in clinical settings being prioritised. With such high student numbers and multiple concurrent classes, an initial audit confirmed variability in debriefing approaches in the simulation labs indicating this aspect of SBE was either inconsistently performed, or without structure and guidance for staff facilitating the debrief.

### The approach to reignite and refine debriefing

To address the situation and gaps in debriefing practices, a two-year funded project commenced late 2022 with the aim of co-designing, implementing, and evaluating a fit-for-purpose debriefing framework. Enabling engagement and input of stakeholders - academic and clinical simulation educators, technical staff, and program leads - was key to addressing local, contextual needs, generating ownership and empowerment in the design process and trustworthiness and adoption of the final ‘product‘ [29]. The muti-stage iterative process described in this paper (Table 1) outlines the steps used in co-designing and implementing a bespoke debriefing framework within an educational setting and offers guidance for others embarking on similar at-scale initiatives.

**Table 1:** – Phases, activities and timeframe within the co-design project based on Wilson et al. (2021)

Phase	Activities	Timeframe
Explore/ideate	Observational audit data as quality improvement; included in grant application Collaborative staff workshops x 3 Review observational audit data; refine questions Several acronyms developed based on common debriefing phases	August – November 2022
Plan/design	SELFIE design agreed upon – initial artwork created Sub-groups formed to work collaboratively on project sections - Staff survey - Focus groups/ interview questions - Other possible applications of the framework Pools of trigger questions developed for each step, based on literature, simulation and educational expertise Regular meetings scheduled for project duration	February – onwards February 2023 – October 2024
Build/test	Staff survey to determine 3 preferred trigger questions for each step Additional questions invited and opinions sought on aspects of debriefing: - Who should lead - Length - Experiences elsewhere Data analysis and updates to the SELFIE tool Educational resources developed - Video clips - Learning management system site - Prompt cards, posters, facilitator guides	March – November 2023
Implement/observe	Pre-implementation workshops for staff SELFIE rolled out in 1 undergraduate nursing course Observation audits continued with refined questions	February – June 2024
Reflect/evaluate	Qualitative questions finalised for student focus groups/interviews; conducted online Post implementation staff survey Data analysis of surveys, focus group/interviews Project report; presentation at university T&L symposium	May & September 2024 September – October 2024 November 2024

### The co-design framework

The co-design approach followed the five phases of the *Connected Learning at Scale* framework [29] to support pedagogic changes in education. The phases included: (1) explore/ideate; (2) plan/design; (3) build/test; (4) implement/observe; and (5) reflect/evaluate. Table 1 provides details of the activities undertaken within each phase of the co-design process, expanded in the following sections.

### Phase 1 - explore/ideate

The project team comprising a number of academic and permanent clinical nurse educators participated in a series of three face-to-face collaborative workshops led by experienced simulation educator/researchers (two of the authors) in late 2022. During these workshops, the history and development of debriefing in healthcare simulation including theoretical and pedagogical approaches were revisited with opportunity for all participants to ideate and contribute to forming a localised framework.

**Fig. 1:**
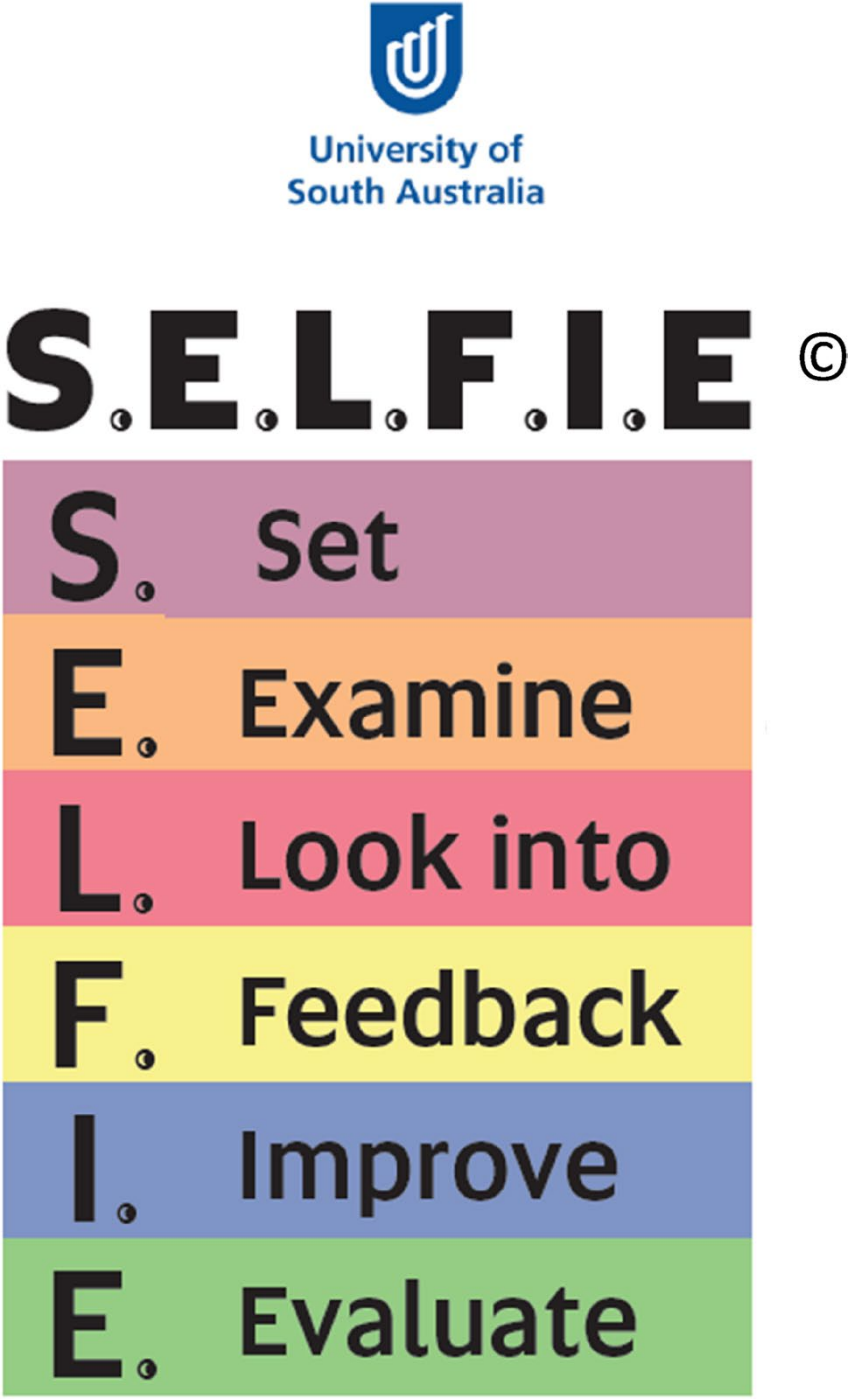
– The SELFIE^©^ framework (short version)

Current practices were discussed within the context of popular or familiar clinical and educational frameworks [30, 31] including the clinical pause concept [32] and a research based model for clinical judgement in nursing [33]. Observational audit data were reviewed with subsequent audits were planned throughout the project to monitor progress and capture changes in practices following introduction of the framework. Some refinement of initial audit questions was done by the core project team to capture more meaningful data going forward. Suggestions emerged about other university teaching contexts where debriefing is or could be used for example following Objective Structured Clinical Assessment/Examination (OSCA/Es) or during student meetings following unsuccessful clinical placements or poor progression within their program of study. Team members brainstormed possible acronyms that would include the core components of debriefing and through an iterative process, the SELFIE^©^ debriefing framework was chosen (Fig. 1).

### Phase 2 – plan/design

Expanding the core components of SELFIE^©^ and creating the initial artwork were next steps (Fig. 1). Sub-groups were formed from the larger project team to work collaboratively on different components. As the project sought evaluative data prior to and following implementation of the framework, university ethics approval was granted (University of South Australia HREC protocol number 205373).

**Fig. 2:**
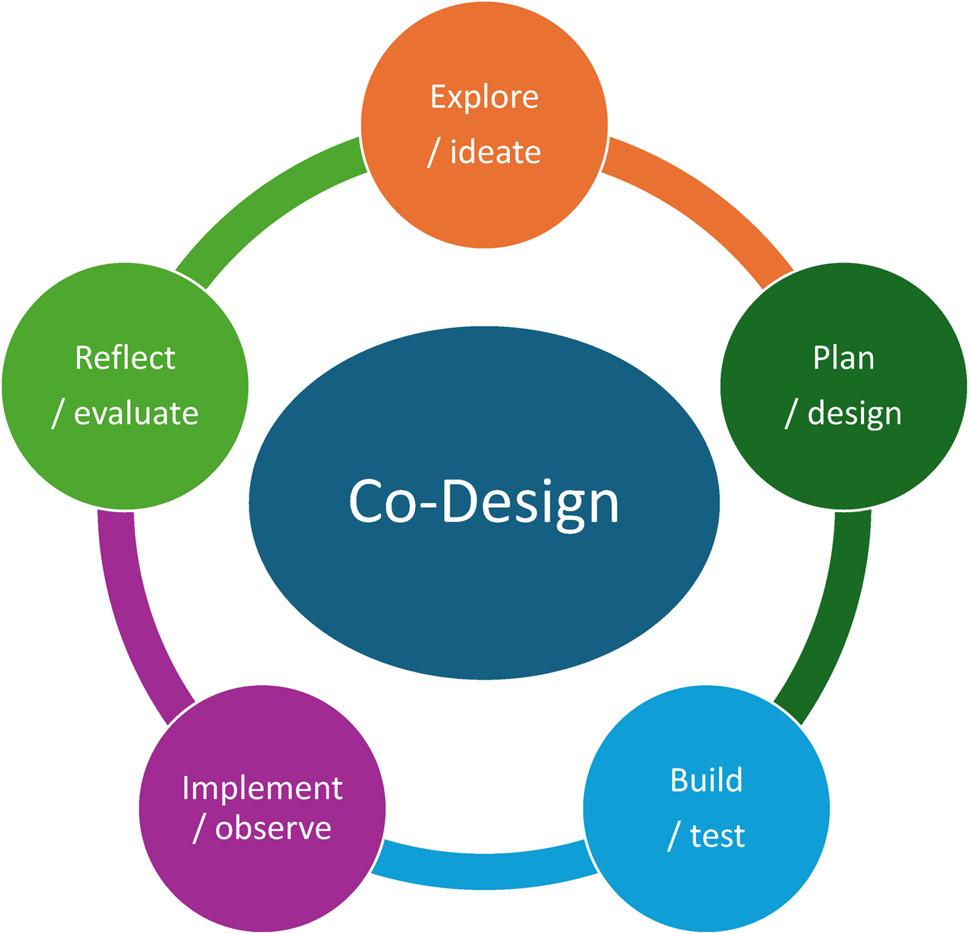
The 5 phases of connected learning at scale co-design framework (Wilson, et al., 2021) applied to creating SELFIE^©^

Pools of trigger questions informed by literature, lived experiences and simulation expertise were created by one sub-group for each of the 6 SELFIE^©^ steps. Another sub-group created an online Qualtrics^XM^ survey for clinical and academic staff comprising initial trigger questions, with opportunity to add further questions, determining the appetite for a new dedicated framework, and preferred supportive strategies prior to implementation. Other sub-group activities were planned for the evaluation phase, and regular online meetings of all project team members were scheduled. These regular meetings provided ongoing opportunity to share perspectives, experiences, field questions aligning with the co-design approach and to keep to schedule (Fig. 2).

### Phase 3 – build/test

#### Pre-implementation staff survey

The pre-implementation Qualtrics^XM^ survey was sent to all academic and clinical educators within the nursing unit to introduce the framework and gain consensus about 3 preferred trigger questions for each of the 6 core SELFIE^©^ steps from the pool of initial trigger questions. Other survey questions invited opinions and preferences on: who should lead the debrief (e.g. faculty team leaders, facilitators, students); the duration and other personal experiences considered beneficial for their own or students’ learning.

#### Refinement of the framework

The practicality of the framework for use at-scale was a key factor. Based on survey feedback, the broader working group achieved consensus on 3 preferred trigger questions to include in each of the 6 framework steps (Table 2). This amount of information had practical intent - to fit into an A4 size prompt sheet and wall posters for easy use and reference. A larger pool of trigger questions for each step was included in the facilitator folders to allow flexibility in the debriefing process and extend reflection and inquiry depending on the level of the learners and educational conext. A range of educational resources was developed in preparation for implementation of SELFIE^©^. Project team sub-groups were given responsibility for:

- creating facilitator guides and posters for use in the simulation labs.
- creating a dedicated site for faculty on the university’s learning management system with a range of additional resources, web links.
- printing small business card size prompt cards for staff and students (Fig. 1).
- working with actors and students to create videos to model application of the framework, including ways to manage different situations (e.g. quiet students, others who overshare information, hesitation particularly with novice students) (see supplementary file of story board).

**Table 2:** SELFIE^©^ framework steps and trigger questions

SELFIE^©^ Six Steps	Examples of trigger questions
S. Set the scene and opening *The first step sets the scene and prepares learners for discussion about what they encountered and involves creating a safe learning environment.*	ϖ How did you (all) feel that went? (venting emotions) ϖ Let’s take some time to talk about what was achieved today ϖ Can a student volunteer talk us through what we learnt today?
E. Examine what went well and challenges *The second step examines in more detail what was familiar or what challenged learners. It also encourages them to draw out or link the simulation to prior experiences.*	ϖ Was there anything you found difficult today? Why or why not? ϖ Has anyone had to deal with this situation before (or undertake these skills) in clinical practice? ϖ What did you notice that you or other students did well today?
L. Look into, focus and discuss key issues *The third step aims to delve deeper and focus on participant concerns to support the learning experience.*	ϖ What was the plan of care, and did the plan change? ϖ Was there anything that made you worried or concerned (during the scenario)? ϖ What were the important patient issues during this scenario?
F. Feedback and translation to practice *The fourth step aims to provide feedback to the participants and connect learning to safe practice*	ϖ How does what you learnt today inform how you will interact with patients? ϖ What is one main thing that you will take away from today’s session? ϖ How does today’s learning relate to safe practice?
I. Improve practice as a result of the debrief *The fifth step encourages the learners to improve practice and apply new knowledge and understanding.*	ϖ How will today’s practice affect what you will do in future sessions and/ or in your clinical practice? ϖ I noticed […], so next time you might want to consider […], because [rationale] ϖ Consider two actions that you would like to work on after today’s session.
E. Evaluate and self-reflect post debrief *The sixth and final step draws together all the learning through evaluating and prompting self-reflection*	ϖ Is there anything you would like clarified or to know more about? ϖ What else can you do to keep improving your practice? ϖ Are there any suggestions on how to remember what you learnt today?

### Phase 4 – implement/observe

#### Planned rollout and SELFIE^©^ pilot testing

A series of pre-implementation professional development sessions was undertaken with SBE teaching staff prior to rollout of the framework in February 2024 in the simulation component of a second year Bachelor of Nursing course. Videos, online resources, prompt cards and facilitator guides were incorporated in these sessions together with opportunity for staff to observe demonstration of the SELFIE^©^ framework, engage in role play and receive feedback on delivery and ways of safely maximising student engagement. In the early stages of the rollout, clinical nurse educators from the project team led and modelled use of SELFIE^©^ within simulation classes, then were present to support casual clinical educators in subsequent classes. Our initial impressions reflected positive uptake of the framework and diffusion of innovation as several of the casual clinical educators taught in multiple courses where they also used SELFIE^©^. Roll out of SELFIE^©^ in other SBE courses within the nursing curricula proceeded with ease. The SELFIE acronym also seemed to resonate with people, as we regularly heard staff and students relate this term to their simulation classes. Observational audits continued, to determine ways the framework was being used and areas for improvement or change.

### Phase 5 – reflect/evaluate

#### Post-implementation staff survey

A slightly modified Qualtrics^XM^ survey, to frame questions in a post implementation inquiry, was distributed to the casual clinical nurse educators and academic staff to gauge uptake and use of SELFIE^©^, end-user experiences and perspectives for ongoing refinement of the framework and if preferred trigger questions had changed. Evaluative data will be reported once fully analysed.

#### Student focus group/interviews

To provide a student perspective on the impact of SELFIE^©^ on learning within simulations, qualitative data were sought. Although focus groups were planned, and one comprising 2 students took place, pragmatically, individual online interviews were more successful as students had completed face-to-face classes, were learning remotely and preparing for end of semester assessments. This aspect of the co-design process, although yielding early insights, provided some perspectives of students’ debriefing experiences prior to and following implementation of SELFIE^©^ and its impact on learning. Findings of all aspects of this project will be presented when finalised.

#### Professional development needs for sustainability at-scale

Noting the large pool of casual clinical educators, regular on-boarding sessions including the use of SELFIE^©^ is an expected ongoing requirement. Our experiences with pre-implementation professional development sessions confirmed that in-person practical workshops, to complement the online resources provided opportunity for staff to rehearse and gain peer feedback on their debriefing practices. Ways of managing and maximising equitable student contribution to debriefing conversations is a common challenge, also raised by our staff. Given the time limits on SBE classes, this is a core focus in staff workshops. Observational audits continued when able, to monitor debriefing practices and determine areas for further development or refinement of the framework.

#### Dissemination

In addition to reporting requirements of the funding body, other opportunities for dissemination of the innovation were planned early in the project and included presentations at the university teaching and learning symposia and relevant conferences, featuring this localised co-designed initiative in a university wide bulletin, and publications in simulation related journals.

#### Expansion of SELFIE^©^ across other university disciplines and learning contexts

As SELFIE^©^ rolled out, word soon spread across the faculty with interest shown in using the framework for simulation in university postgraduate nursing and midwifery programs and medical radiation. During subsequent professional development sessions, casual nurse educators facilitating students clinically, expressed interest in using SELFIE^©^ as a framework to structure feedback to students on their practice. With additional questions to draw from in each of the six core SELFIE^©^ steps, the flexibility and applications of the framework for other university learning activities beyond simulation was becoming evident.

### Discussion

There is general consensus amongst the healthcare simulation community that debriefing is an essential component of these experiential learning activities [1]. Although several debriefing frameworks exist, these need to be relevant for use in different contexts, settings, and disciplines mindful of learner and faculty cultural nuances, and be suitable for use at-scale [10, 20, 34]. In addition, we perceived multiple benefits in using a co-design approach to create a fit-for-purpose localised debriefing framework. In our university nursing program, leveraging off educational theoretical perspectives, other debriefing frameworks and staff’s lived experiences helped us reach an agreed framework that would resonate with staff, and one that would be adopted with pride.

The SELFIE^©^ debriefing framework was developed to address a gap in simulation delivery at our institution to reinstate a pragmatic way of debriefing given the challenges of providing SBE at-scale to diverse student cohorts. The co-design approach to creating the framework actively engaged the faculty living the change, promoting a sense of ownership, increased receptiveness and a willingness to engage in new practices [28]. The flexibility of our framework, with the 6 core steps and a wider pool of alternatively framed trigger questions for each step, allows a degree of nuance to match the learning situation at hand. In addition to using the framework within SBE, other potential uses in the educational context include: during student clinical practice experiences or university theoretical tutorials, for remediation following unsatisfactory student assessments, or when counselling students.

While the SELFIE^©^ debriefing framework was co-designed and implemented to guide, improve, and develop debriefing practices within the university simulation learning environment, it is not intended as a fully prescriptive tool. The design of SELFIE^©^ allows for facilitator flexibility, such as merging one or two of the core steps or moving forward or back to other steps in response to the learners’ input. Given our challenges of large student cohorts with varying learner needs, and pool of casual clinical educators, we believe that SELFIE^©^ is a particularly useful tool for novice faculty learning how to facilitate debriefing in university nursing programs, while providing consistency at-scale across SBE classes.

### Lessons learned

Co-design was a rewarding and worthwhile approach for this project however involving multiple stakeholders presented pragmatic issues that can delay progress but ultimately enrich the outcome. Providing opportunity for stakeholders to be involved, to afford voice and perspective in the framework design of ‘what matters to me’ [28], resulted in shared ownership of the outcomes and motivation to implement a change in practice. When involving students, you need to consider the best strategies to have them invested in the process, and to be more willing to be a part of the input.

## Conclusion

The co-designed fit-for-purpose SELFIE^©^ debriefing framework, created to address a gap in at-scale simulation practices at our university, proved a successful endeavour with broad uptake and diffusion of this innovation. The co-design approach was central to the development of SELFIE^©^ to ensure and support best teaching practices through ownership of the ‘end product’. While developed for nursing SBE, adaptability and flexibility of the framework for a variety of higher education settings and contexts has already emerged. Future activities will focus on evaluating the impact of the SELFIE^©^ framework longitudinally on our students’ learning and practice, and ways other health student disciplines may apply the framework. Feasibility, applicability and relevance of the framework, for example in university based interprofessional SBE, would contribute to further refinements.

## Supplementary Information

Supplementary Material 1.

## Data Availability

No datasets were generated or analysed during the current study.
