# Supplementary material for: Using co-design to create a fit-for-purpose debriefing framework for at-scale healthcare simulation
Source: Adv Simul (Lond). 2026 Apr 30;11:49. doi: 10.1186/s41077-026-00441-y (PMC13335274; doi:10.1186/s41077-026-00441-y)
Supplement: Supplementary file 1 — Supplementary Material 1. [file 41077_2026_441_MOESM1_ESM.docx]

| **Background to story**  The Stage 2 nursing students have just finished a couple of activities in the HHHS where they had to problem solve and undertake a clinical assessment of a patient.   1. Patient has just returned from theatre, on oxygen therapy, lying flat with one pillow and rings the call bell because feeling nauseated. The students will need to assess the patient, reposition and check the medication chart. 2. Four hours later, following internal fixation of a fractured fibula, patient rings the bell because they have pain and are thinking about having to mobilise to the toilet. 3. However, the patient does not speak very good English and have trouble explaining that they have pain. The students will need to assess, reposition and check the medication chart.   Skills needed (Heath of Adults)  (respiratory assessment, nausea and vomiting medication, fractures, post-operative care)  (pain assessment and options for mobilisation)  (communication with patient)  (problem-solving)  (medication management) |
| --- |

Narrator: We’re going to illustrate how a facilitator might use the SELFIE debriefing framework in each of the 6 steps. This video is specifically geared towards HHHS sessions. The prompts can be modified – using different words – but the intent is to draw out learners’ thoughts and guide the reflective process. Not all prompts need be used – adapt for the situation and context.

The 1st step is S = Put on up screen

**S. Set the Scene & Opening**

- **How did you (all) feel that went? (venting emotions)**
- **Let’s take some time to talk about what was achieved today.**
- **Can a student volunteer to talk us through what we learnt today?**

| **1.**  **Set the Scene**  (Students are to gather around a bedside, getting ready to debrief.)  Facilitator: “Okay, so is everyone ready to have a chat about what you achieved in your HHHS session today?  Students nod, say yes etc…  Facilitator: Overall, how did you think that went?”  Students mumble quietly …  Facilitator: “Does someone want to start us off and recap what our learning was focused on today?”  Student 1: Well today we had a patient who had just returned to the ward following surgery on their leg, and we had to give them something for their nausea…….  Student 2: and then we had to do a pain assessment, but the patient did not speak very good English, and it was hard to work out what they were saying….  Student 3: And they wanted to go to the toilet, and I wasn’t sure what they should do …  Facilitator: OK – let’s unpack that a bit more …  Fade out. Pause |
| --- |

Narrator: To recap – this 1^st^ step sets the scene and prepares learners for discussion about what they encountered

Put on up screen **S. Set the Scene & Opening**

- **How did you (all) feel that went? (venting emotions)**
- **Let’s take some time to talk about what was achieved today.**
- **Can a student volunteer to talk us through what we learnt today?**

Narrator: now that the facilitator has set the scene, we move on to the 2^nd^ step E: examining what played out in the scenario

Put on up screen **E. Examine what went well & challenges**

- **Was there anything you found difficult today? Why or why not?**
- **Has anyone had to deal with this situation before (or undertake these skills) in clinical practice?**
- **What did you notice that you or other students did well today?**

| **2.**  **Examine what went well and challenges**  Facilitator: “I want you to remember back to … any … of your clinical placements – did anyone experience a similar situation to what we’ve worked through today?”  Students nod  Facilitator: “Jasmine do you want to tell us what happened in that situation – and was it similar to this? And what was your role?”  Student 1: Yes well … when I was on my aged care placement, we were told to ask residents how they were, if they had any pain, and usually in the morning before we started their showers and ADLs.  Sometimes they had trouble explaining their pain……I had one resident who had just had and stroke and…..” (fade out)  Facilitator: “Yes that is important to check in regularly with those you’re caring for, in your case the residents.  That way you can prevent them having to worry about how they are going to get up and about, but then you can think about the type of questions to ask, to accurately assess their pain”  “Any other students have similar situations?”  No answer  Facilitator :“Was there anything that you found difficult today?”  Students nod.  Student 2: “I found it hard to know if I could, so when the patient has just had surgery for a fractured fibula …. (fade out) |
| --- |

Narrator: To recap – this 2^nd^ step examined in more detail what was familiar or what challenged learners – and to draw out or link their prior experiences.

Put on up screen

**E. Examine what went well & challenges**

- **Was there anything you found difficult today? Why or why not?**
- **Has anyone had to deal with this situation before (or undertake these skills) in clinical practice?**
- **What did you notice that you or other students did well today?**

Narrator: The 3^rd^ step – L = looks into, delves deeper, and focuses on any concerns to support the learning experience

Put on up screen

**L. Look into, focus and discuss key issues**

- **What was the plan of care, and did the plan change?**
- **Was there anything that made you worried or concerned (during the scenario)?**
- **What were the important patient issues during this scenario?**

| **3.**  **Look into focus and discuss key issues**  Facilitator: “So Jasmine spoke about the resident who had a stroke and was unable to verbalise so they had to use a visual analogue scale. But in today’s scenario what was it that made it challenging to undertake your pain assessment?  Student 1– “I think knowing you might not always know when a patient is telling you they have pain”  Facilitator: “that’s a good point, xxxx, I think through more experiences, you will all become more familiar with how patients might react to pain … knowing there are different ways for a patient to show you they’re in pain”.  Students nod and ‘oh …’.  Facilitator: “Now what do you think was the most important issue in this scenario”?  Student 2 – “well I would say the main thing was that we needed to assess their pain so that we could work out how to manage it”  Facilitator: “excellent – we can link that back to our objectives of the session today which was to be able undertake a pain assessment of a post-operative patient”  Facilitator: “now remember this patient had only just returned from theatre having had orthopaedic surgery. What other reasons might be causing pain following this procedure?  Fade out… |
| --- |

Narrator: To recap, this 3^rd^ step aimed to delve deeper, and focus on learner concerns going forward

Put on up screen

**L. Look into, focus and discuss key issues**

- **What was the plan of care, and did the plan change?**
- **Was there anything that made you worried or concerned (during the scenario)?**
- **What were the important patient issues during this scenario?**

Narrator: The 4^th^ step – F – moves to the stage of providing feedback and linking learning to safe clinical practice.

Put on up screen

**F. Feedback & translation to practice**

- **How does what you learnt today inform how you will interact with patients?**
- **What is one main thing that you will take away from today’s session?**
- **How does today’s learning relate to safe practice?**

| **4.**  **Feedback and translation to practice**  Facilitator: “Let’s find out how what was learnt today can be applied to clinical practice. Who wants to start? What is one thing you will take away from the session today and apply to your practice?”  Student 1: “Well, I learnt that it is a good idea to know how you would do a pain assessment when a patient can’t express their pain.”  Student 2: “yes, good one. And for me, today made me realise it’s important to take off the oxygen mask BEFORE the patient vomits and try to reposition them to catch the vomit in the bag ….”  Facilitator: that’s a really important point don’t you think? Prevents them from aspirating?  Student 3: “ah yeah! …and to also reassure them that we’ll get some medication to help reduce the nausea “  Fade out … |
| --- |

Narrator: To recap – this 4^th^ step aimed to provide feedback and connect learning to safe practice.

Put on up screen **F. Feedback & translation to practice**

- **How does what you learnt today inform how you will interact with patients?**
- **What is one main thing that you will take away from today’s session?**
- **How does today’s learning relate to safe practice?**

Narrator: The 5^th^ step - I - encourages the learner to improve practice, and apply new knowledge and understanding.

Put on up screen

**I. Improve practice** **as a result of the debrief**

- **How will today’s practice affect what you will do in future sessions and/ or in your clinical practice?**
- **I noticed […], so next time you might want to consider […], because [rationale]**
- **Consider two actions that you would like to work on after today’s session.**

| **5.**  **Improve practice as a result of the debrief**  Facilitator: “Thanks everyone for sharing what you learned out of today’s session. “  “I noticed that most of you picked up on the need for repositioning a patient when they are feeling nauseated… but some of you were worried about repositioning this patient due to the type of surgery they had…”  Students nod.  Facilitator: “So next time you might want to consider putting preventative measures in place if you are anticipating that nausea and vomiting is common after general anaesthetic. For example, you can think about safe positioning, antiemetics and so forth…”  Students nod. Yeah, really important …  Facilitator: “So, what are some things you might want to consider now, after today’s session?”  Student 1: “well, I’m going to go back and refresh my medication knowledge about antiemetics.”  Student 2: “I’m going to have a better look at the medication charts, so I know where to find where the antiemetics are charted.”  Student 3: “I want to know more about the side effects of general anaesthetics…” |
| --- |

Narrator: The 5^th^ step encouraged the learner to apply new knowledge and understanding in order to improve practice.

Put on up screen **I. Improve practice as a result of the debrief**

- **How will today’s practice affect what you will do in future sessions and/ or in your clinical practice?**
- **I noticed […], so next time you might want to consider […], because [rationale]**
- **Consider two actions that you would like to work on after today’s session.**

Narrator: The 6^th^ and final step - E – draws together all the learning through evaluating and prompting self-reflection.

Put on up screen **E. Evaluate & self-reflect post debrief**

- **Is there anything you would like clarified or to know more about?**
- **What else can you do to keep improving your practice?**
- **Are there any suggestions on how to remember what you learnt today?**

| **6.**  **Evaluate and self-reflect post debrief**  Facilitator: “Let’s wrap this up for today… and thanks everyone for your participation. Are there are any other areas from today that you would like clarified or to know more about?”  Student 1: “As a nurse, is it our job to know about ALL OF the medications? I just feel like there are a lot to know….”  Facilitator: “There are a lot of medications to know, and it is really important that you have a good understanding of antiemetics, for example, so you can predict your patients’ needs….” (fade out)  Facilitator: “Are there any suggestions on how to remember what you learned today?”  Student 2: “I have a notebook of drugs that I take notes on as I come across them….. “(fade out)….  Student 3: and I’ve got an app to look up the medications we covered today … (fade out)….  Facilitator: “great discussions, thanks everyone for participating. We’ve now come to the end of our time, but if you think about more things you’d like to discuss, let’s connect next week …” |
| --- |

Narrator: To recap, this 6^th^ and final step - E – brought together all the learning through evaluating and prompting self-reflection.

Put on up screen

**E. Evaluate & self-reflect post debrief**

- **Is there anything you would like clarified or to know more about?**
- **What else can you do to keep improving your practice?**
- **Are there any suggestions on how to remember what you learnt today?**

Narrator: We’ve pulled together all aspects of the SELFIE debriefing framework here and illustrated ways the facilitator can guide learners’ thinking, reflection and application of learning to practice.

Put on up screen the whole SELFIE figure
